# Efficient activation of gene expression using a heat-shock inducible Gal4/Vp16-UAS system in medaka

**DOI:** 10.1186/1472-6750-4-26

**Published:** 2004-10-26

**Authors:** Clemens Grabher, Joachim Wittbrodt

**Affiliations:** 1 Developmental Biology Program, European Molecular Biology Laboratory (EMBL), 69117-Heidelberg, Germany

## Abstract

**Background:**

Genetic interference by DNA, mRNA or morpholino injection is a widely used approach to study gene function in developmental biology. However, the lack of temporal control over the activity of interfering molecules often hampers investigation of gene function required during later stages of embryogenesis. To elucidate the roles of genes during embryogenesis a precise temporal control of transgene expression levels in the developing organism is on demand.

**Results:**

We have generated a transgenic Gal4/Vp16 activator line that is heat-shock inducible, thereby providing a tool to drive the expression of specific effector genes via Gal4/Vp16. Merging the Gal4/Vp16-UAS system with the I-SceI meganuclease and the *Sleeping Beauty *transposon system allows inducible gene expression in an entirely uniform manner without the need to generate transgenic effector lines. Combination of this system with fluorescent protein reporters furthermore facilitates the direct visualization of transgene expressing cells in live embryos.

**Conclusion:**

The combinatorial properties of this expression system provide a powerful tool for the analysis of gene function during embryonic and larval development in fish by ectopic expression of gene products.

## Background

The most widely used strategies to investigate the function of genes in medaka (*Oryzias latipes*) are the analyses of mutants, miss-expression of wild type genes or their variants by mRNA injection and gene specific translational inhibition by morpholino injections [1-3]. However, the phenotype of a given mutation mainly reflects the first temporal function of the affected gene in embryonic development, obscuring possible later functions. Similarly, mRNA and morpholinos exert their functions immediately following injection, providing information only on the early role of the gene of interest. A detailed analysis of gene function in a given process can thus be a difficult task. The Gal4/UAS system provides an alternative and more specific strategy to analyze specific functions of a gene [4,5]. The direct application of the *Drosophila *Gal4-UAS approach, by the generation of transgenic lines, has been established successfully in zebrafish [6,7]. However, the generation of different transgenic activator and effector lines may be a time- and space-consuming task, and expression levels in these transgenic lines are weak, probably due to a limited transactivation potential of Gal4 in fish. Gal4/Vp16, a fusion of the yeast Gal4 DNA-binding domain with the strong Vp16 transactivation domain of the herpes simplex virus [8] can be used to enhance transactivation efficacy. Yet, strong transcriptional activators can cause unspecific promoter squelching [9] resulting in retardation of embryogenesis [10]. Nonetheless, the Gal4/Vp16-UAS system has been used in zebrafish in transient approaches resulting in mosaic, but easily detectable transgene expression [11].

We have applied the Gal4/Vp16-UAS system for transient transactivation in a heat-shock inducible transgenic Gal4/Vp16 activator line. Generation of transgenic medaka lines, which allow the induction of the Gal4/Vp16 activator to 'physiological' (i.e. non-toxic) levels was achieved by using a 5' truncated version of the zebrafish heat-shock promoter HSP70 [12]. Using a heat-shock promoter to drive expression of the Gal4/Vp16 activator allows tight temporal control of activator and effector (reporter) gene expression. To trace transgene expression in cells of living embryos we have used the cyan fluorescent and yellow fluorescent proteins (CFP, YFP). Combination with the meganuclease (MN) transgenesis system [13] and the direct-inverted repeats (IR/DR) of the *Sleeping Beauty *(SB) transposon system [14] yielded high numbers of transgene expressing cells. Thus, in contrast to the entirely mosaic nature of a transient approach reported thus far, the combined use of a transgenic activator line with systems enhancing even DNA distribution or early integration allows uniform expression of injected effector genes upon induction by heat-shock treatment without an immediate need to generate transgenic UAS lines.

## Results and discussion

### Generation of a heat-shock inducible transgenic Gal4/Vp16 activator line (pCG6.0WCS/T)

**Figure 1:**
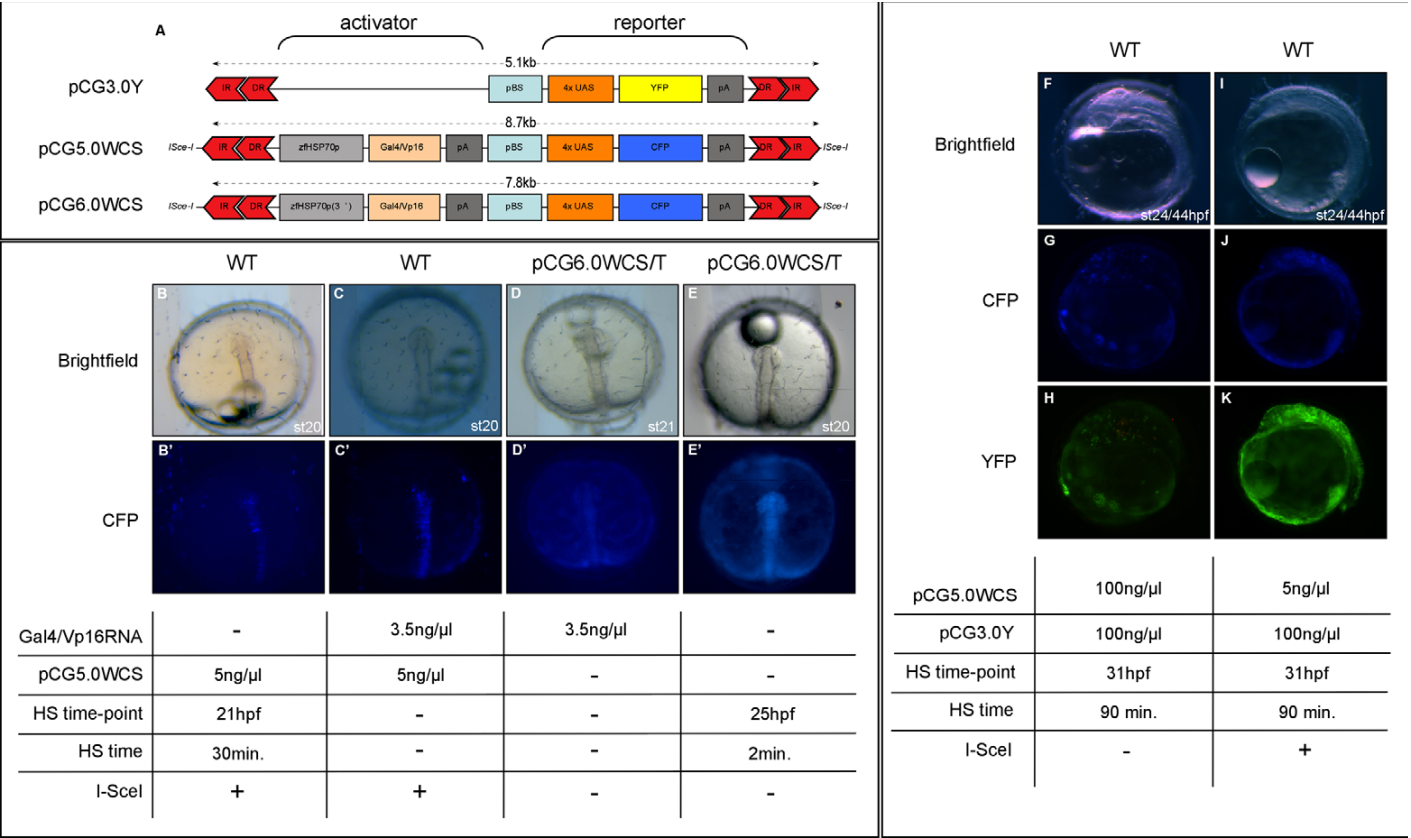
**Schematic representation of Gal4/Vp16-UAS expression vectors (A), comparison of induction levels between transient and transgenic approaches (B-E') and transient Gal4/Vp16 mediated reporter expression in medaka embryos induced by heat-shock (F-K). **A; Gal4/Vp16 activator units driven by a 1.5 kb (pCG5.0WCS) or a 600 bp (pCG6.0WCS) heat-shock promoter fragment followed by a SV40 polyadenylation signal are shown on the left. Reporter units are separated from the activator units by the pBSII backbone. YFP (pCG3.0Y) or CFP (pCG5.0WCS/6.0WCS) open reading frames are placed downstream of 4 UAS elements and followed by a SV40 polyadenylation signal. Entire expression cassettes are flanked by the IR/DRs of the SB transposon system. Additionally, I-SceI meganuclease sites flank the expression cassettes of activator vectors. Abbreviations and actual sizes of each vector are given. B-E'; Wild type medaka embryos were injected with pCG5.0WCS and MN and subjected to heat-shock treatment (B, B') or with MN and Gal4/Vp16 mRNA without treatment (C, C'). Similarly, the transgenic activator line pCG6.0WCS/T was injected with Gal4/Vp16 mRNA (D, D') or subjected to heat-shock treatment (E, E'). Anterior is to the top (B-E'). DNA and RNA concentrations are indicated together with the developmental stage and the duration of heat-shock treatment. Microinjection of the activator/reporter plasmid pCG5.0WCS results in activation of CFP according to the distribution of plasmid DNA (B-C'). In contrast, induction of activator and reporter in the transgenic line by mRNA injection or heat-shock treatment results in ubiquitous and entirely uniform expression of CFP (D-E'). F-K: Activator and reporter vectors were injected into one-cell stage medaka embryos. Anterior is to the left (F-K). Developmental stage at heat-shock induction and duration of treatment is indicated. HS treatment of up to 2 hours did not interfere with embryonic development but yielded detectable transgene expression (F-K). CFP (internal reporter) shows the expression pattern of the activator Gal4/Vp16. YFP shows activation of the independent reporter. Co-injection of activator pCG5.0WCS and independent reporter pCG3.0Y (100 ng/μl each) resulted in mosaic activation of reporter gene expression (F-H) only. Co-injection of activator pCG5.0WCS (5 ng/μl) and independent reporter pCG3.0Y (100 ng/μl) with I-SceI resulted in a broad range of different levels of mosaicism. Notably, 16% of co-injected embryos showed highly uniform expression (I-K). Abbreviations: CFP, cyan fluorescent protein; HS, heat-shock; hpf, hours post fertilization; IR/DR, inverted/direct repeats; MN, meganuclease; pA, SV40 polyadenylation signal; pBS, pBluescriptII; st, developmental stage; UAS, upstream activating sequence; zf, zebrafish.

DNA injection leading to mosaic expression in G0 allows *in vivo *tracing of transgene-expressing cells and observation of effects exerted by the transgene through application of fluorescent markers [11]. However, elucidation of biological questions sometimes requires ubiquitous expression of transgenes in a temporally controlled manner. While the MN protocol strongly reduces mosaicism, it does so only in a fraction of injected embryos ([13], Fig. 1F,1G,1H,1I,1J,1K and Table 1). This can be improved by the use of transgenic animals providing inducible and sufficient expression in all cells.

The idea is to combine stable heat-shock inducible expression of the Gal4/Vp16 activator with transient expression of effector genes upon microinjection. The effector constructs are uniformly distributed in the entire embryo due to the presence of the SB direct-inverted repeats [15]. We have designed two activator/reporter vectors containing Gal4/Vp16 under control of a 1.5 kb fragment of the zebrafish (zf) HSP70 promoter (pCG5.0WCS) or a 0.6 kb 5' truncated fragment of zfHSP70, respectively (pCG6.0WCS). Both vectors contain CFP downstream of several UAS elements as an internal reporter. The IR/DRs of SB and two I-SceI meganuclease sites flank this entire expression cassette (Fig. 1A). The internal reporter provides a direct read-out for activator expression. A third vector (pCG3.0Y), containing YFP downstream of several UAS elements and flanked by IR/DRs, was designed as an independent reporter (Fig. 1A).

It has been shown that the Gal4/Vp16 activator can interfere with general transcription by titrating the basal transcription machinery [16]. We observed developmental retardation and malformation in all embryos injected with Gal4/Vp16 driven by ubiquitous promoters. Similarly, co-injection of high concentrations of Gal4/Vp16 mRNA (50 ng/μl) with pCG5.0WCS always resulted in developmental malformations (not shown). However, DNA co-injections did not affect embryonic development in transient experiments when the HSP70 promoter was used to control Gal4/Vp16 expression (Fig. 1B,1B',1F,1G,1H,1I,1J,1K). Moreover, co-injections of low concentrations of Gal4/Vp16 mRNA (3.5 ng/μl) with the activator/reporter construct pCG5.0WCS also showed no effects on embryogenesis (Fig. 1C,1C'), suggesting that the toxicity of the activator depends on the expression level.

The truncated version of the zfHSP70 promoter fragment used in the activator/reporter construct pCG6.0WCS showed a moderate activation upon heat-shock treatment. This allowed adjusting the induction levels by varying the heat-shock duration. A transgenic medaka line was established (by co-injection of circular vector pCG6.0WCS with MN) in which expression levels directly correlated with the heat-shock duration. Extended heat-shocks resulted in very high expression levels, but also caused retardation phenotypes due to the strong transactivation potential of the Gal4/Vp16 fusion protein. Comparable phenotypes were not observed in heat-shock treated wild type embryos. Depending on the developmental stage at the time of induction, the duration of heat-shock treatment was adjusted to induce Gal4/Vp16 and reporter expression without interfering with embryonic development. Induction periods ranged from one minute of heat-shock at 37°C at early stages (~st16/21hpf) to 10 minutes at later stages (~st22/38hpf). On top of uniform CFP expression in the entire embryo and yolk upon heat-shock, some regions of the embryo showed additional responsiveness of the reporter (Fig. 1E,1E').

Microinjection experiments and RT-PCR revealed that reporter gene (CFP) expression in transgenic fish is mediated by Gal4/Vp16. Offspring of pCG6.0-WCS/T transgenic fish was injected with Gal4/Vp16 mRNA (3.5 ng/μl) at the one-cell stage without heat-shock treatment. Injected embryos exhibited uniform expression of CFP shortly after the onset of zygotic transcription at the mid-blastula transition [17], indicating that CFP expression was induced in response to Gal4/Vp16 (Fig. 1D,1D').

**Figure 2:**
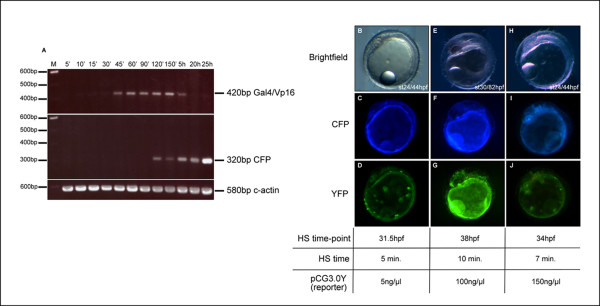
**Kinetics of activator and reporter induction (A) and activation of an independent reporter in pCG6.0WCS/T (B-J). **A; Transcriptional induction of the activator Gal4/Vp16 and the internal reporter (*CFP*) in pCG6.0WCS/T was analyzed by RT-PCR. Embryos were heat-shock for 90 seconds at st20/31.5hpf and were allowed to recover for the indicated periods of time. Activator transcripts were detectable already 10 minutes after induction and the levels increased up to 3 hours. Degradation to undetectable levels was complete after 20 hours. Activator dependent transcription of the internal reporter CFP was observed only after 2 hours and levels were still increasing after 25 hours. C-actin was used as an internal control. (B-J); The independent reporter plasmid pCG3.0Y was injected into the transgenic Gal4/Vp16 activator line. Anterior is to the left (B-J). Expressions of both internal and independent reporters were observed in a weak to strong ubiquitous manner (B-J). Occasional higher levels of internal reporter expression were observed in some parts of the embryonic body. These higher levels were paralleled by independent reporter expression (B-D, H-J). Mosaic clones exerting stronger YFP expression reflect locally higher plasmid concentrations (B-J). Abbreviations: h, hours; hpf, hours post fertilization; HS, heat-shock; M, size marker; st, developmental stage.

We applied RT-PCR for the dose/response analysis of activator and reporter mRNA (Fig. 2A). Transcripts of Gal4/Vp16 were detectable already 10 minutes after a heat-shock of 90 seconds at 37°C. Following a steady increase until about three hours after induction, Gal4/Vp16 messages were degraded between five and ten hours to undetectable levels after twenty hours. CFP mRNA was first detected after two hours and transcript levels were still increasing after 25 h. This indicates that the transcription of the reporter CFP is controlled by Gal4/Vp16 protein and that active Gal4/Vp16 is still present when the amount of its transcripts already dropped below detectable levels (Fig. 2A).

### Activation of an independent reporter upon injection into the transgenic activator line pCG6.0WCS/T

We tested the Gal4/Vp16 activator line pCG6.0WCS/T as a tool to induce expression of an independent reporter upon injection of plasmid DNA (pCG3.0Y). Transgenic embryos were injected with different concentrations of the reporter pCG3.0Y (5–150 ng/μl). Injected embryos were subjected to heat-shock treatment at different developmental stages for various periods of time, kept at 28°C thereafter and monitored for activator and effector expression during the following days. Due to the SB IR/DRs flanking the expression cassette, the independent reporter was distributed equally in the entire embryo resulting in ubiquitous expression of YFP, entirely co-localizing with the internal reporter (CFP). Additional mosaic clones of cells expressing YFP at higher levels presumably reflect higher plasmid concentrations in these cells (Fig. 2B,2C,2D,2E,2F,2G,2H,2I,2J and Table 1). However, YFP expression levels appeared relatively independent from the DNA concentration, but were directly correlated to the expression levels of the activator or internal reporter, respectively.

## Conclusions

Here we show that the Gal4/Vp16-UAS transactivation system can be efficiently used in medaka. By using fluorescent proteins as internal or independent reporter, cells co-expressing the activator and the gene of interest can be visualized directly. Transparency of these fish embryos allows the evaluation of the cellular fate and response to ectopically expressed genes by time-lapse analyses. The combination with inducible promoters permits temporal control of effector gene expression and enables the modulation of the response intensity by adjusting the duration of the heat-shock treatment. This inducible system can be used in transient experiments to study the behavior of transgene expressing cells in an otherwise wild type environment. The combination with the MN and SB system offers to tailor a range of different levels of mosaicism (Fig. 1F,1G,1H,1I,1J,1K). A transgenic Gal4/Vp16 activator line was generated, which provides a powerful tool to induce activator and effector gene expression in a ubiquitous manner at a given time-point (Fig. 1E,1E'). When used in microinjection approaches of reporter vectors containing IR/DRs, our transgenic activator line allows ubiquitous and uniform expression of the reporter gene without the need to generate transgenic effector (UAS) lines (Fig. 2B,2C,2D,2E,2F,2G,2H,2I,2J). In addition to temporal control mediated by the heat-shock promoter, induction using a focused laser-beam [12] could provide precise spatial control of the effector gene expression.

## Methods

### Plasmids

#### pCG3.0Y

A YFP/SV40pA cassette was cloned downstream of a 4xUAS/dHSP70 element (non responsive to heat-shock; kind gift of M. Gonzalez-Gaitan). This entire cassette was cloned into pCG1.1 containing the IR/DR sequences of the SB transposable element [14] resulting in a 5.1 kb plasmid containing the reporter cassette (4xUAS/dHSP70/YFP/SV40pA) and the pBSII backbone flanked by a left and right IR/DR of SB (Fig. 1).

#### pCG5.0WCS

A 1.5 kb zebrafish HSP70 promoter fragment [12] was subcloned upstream of Gal4/Vp16/SV40pA. The entire cassette was further subcloned into pCG3.0C (containing CFP instead of YFP, see above) resulting in a 8.7 kb plasmid (pCG5.0C) containing the expression cassettes zfHSP70/Gal4/Vp16/SV40pA followed by 4xUAS/CFP/SV40pA flanked by the IR/DRs of SB. Finally, the expression cassettes including the inverted repeats were cloned into a I-SceI backbone vector [13] and verified by sequencing (Fig. 1).

#### pCG6.0WCS

The 1.5 kb zfHSP70 promoter fragment in pCG5.0WCS was replaced by a truncated zfHSP70 promoter fragment lacking 900 bp 5' to the internal *BamHI *site resulting in a 7.8 kb plasmid that was verified by sequencing (Fig. 1). Further structural details of the activator and reporter vectors are available upon request.

#### pCGGal4/Vp16

A Gal4/Vp16 fusion construct (Gal4 DNA binding domain: amino acids (aa) 1–147 and Vp16 transactivation domain: aa 411–491) was designed from Clontech vectors pM (Gal4) and pM3-VP16 (Vp16) and cloned into pCS2+ [18].

Gal4/Vp16 mRNA was transcribed *in vitro *from pCGGal4/Vp16 using the mMessage mMachine kit (Ambion Inc.).

### Microinjection and heat-shock treatment of medaka embryos

For microinjections, one-cell stage embryos of the Cab inbred strain were used. Microinjection capillaries were backfilled with the injection solution [DNA (5–150 ng/μl); Yamamoto buffer (1×) or DNA (5 ng/μl); Yamamoto buffer (0.5×); I-SceI buffer (0.5×, New England Biolabs); I-SceI meganuclease (0.35 u/μl, New England Biolabs) with or without Gal4/Vp16 mRNA (3.5–50 ng/μl)]. DNA was prepared using a Qiagen Maxiprep kit (Qiagen, USA) and dialyzed using nitrocellulose filters (#VSW01300; Millipore, USA). DNA was injected through the chorion into the cytoplasm of one-cell stage embryos. Heat-shock treatment was performed in small volumes (100–200 μl) using a waterbath at 37°C. Animals used in the study were kept according to national and international ethical provisions for animal husbandry as implemented at EMBL.

### Microscopy

Embryos were observed and scored using a MZFLIII dissecting microscope with a 436/20 nm (EF); 480/40 nm (BF) filter set for CFP, a 510/20 nm (EF); 560/40 nm (BF) filter set for YFP and a 360/40 nm (EF); 420 nm (BF) filter set for UV/Brightfield. The stereomicroscope was equipped with a DC500 digital camera for imaging (Leica Microsystems, Germany).

### RNA isolation and RT-PCR

Transgenic embryos were heat-shocked and subsequently kept at 28°C to recover for different periods of time. Total RNA was isolated from individual embryos as described [19]. Total RNA was subjected to reverse transcription (SuperscriptII, Gibco-BRL) using a mixture of random hexamer primers (25 μM, Amersham) and gene specific oligomeres for Gal4/Vp16 (25 μM; 5'-CCACGTCCAAAGCCCCATAC-3') and CFP (25 μM; 5'-GTTCATCCATGCCATGTGTAATCCC-3') in a 20 μl reaction. 2 μl of each RT reaction was used for PCR in a 50 μl reaction. The primer pairs used were Gal4/Vp16up2 (5'-GATAATGTGAATAAAGATGCCGTCA-3') and Gal4/Vp16low2 (5'-CCACGTCCAAAGCCCCATAC-3') to amplify a 420 bp fragment, and CFPup2 (5'-TCAAGGAGGACGGCAACATC-3') and CFPlow2 (5'-GTTCATCCATGCCATGTGTAATCCC-3') to amplify a 320 bp fragment. Amplification of a 580 bp fragment of c-actin was used as an internal control using the intron-spanning primer pair c-actinup2 (5'-GCCGCGACCTTACAGACTACCT-3') and c-actinlow2 (5'-CTGTTTAGAAGCATTTGCGGTGGAC-3'). An initial 1 min. denaturation step at 95°C was followed by an additional denaturation step for 30 sec. at 95°C, annealing for 30 sec. at 60°C and elongation at 72°C for 30 sec. The program was repeated for 30 cycles followed by a final extension step for 5 min. at 72°C.

## Authors' contributions

CG designed and performed all experiments and drafted the manuscript. JW conceived of the study, and participated in its design and coordination. All authors read and approved the final manuscript.

## Figures and Tables

**Table 1:** Microinjection of reporter vector.

				Reporter expression		
				
Vector	MN	Host	Fish	before induction^1^	after induction^2 ^(mosaic)	after induction^2 ^(uniform)
pCG5.0WCS pCG3.0Y	+	wildtype	74	52	61/61 (82%)	12/12 (16%)
pCG3.0Y	-	pCG6.0WCS/T^3^	162	29	-/-	73/73 (100%)

1...Leaky expression was mainly observed in the yolk only

2...Expression of internal (CFP) and independent (YFP) effector in the embryonic body was scored separately; CFP/YFP

3...Injection was performed in offspring of heterozygous carriers mated to wildtype fish, resulting in ~50% transgenic embryos
